# Empowering Students to Identify Their Own Skill Sets Through a Final Year Biomedical Science Job Interview Assessment

**DOI:** 10.3389/bjbs.2025.14887

**Published:** 2026-02-05

**Authors:** Kayleigh Wilkins, Amreen Bashir, James Heritage, Kathleen Pritchard, Ross Pallett, Karan Singh Rana

**Affiliations:** School of Biosciences, College of Health and Life Sciences, Aston University, Birmingham, United Kingdom

**Keywords:** biomedical science, graduate careers, higher education, job interview skills, undergraduate curriculum

## Abstract

**Introduction:**

The final year Professional Development for Biomedical Scientists module at Aston University strives to create competent practitioners upon graduation. Recent research identified that 93% of NHS pathology employers within the United Kingdom, do not believe that new Biomedical Scientist graduates possess the skills required for a Band 5 interview. Additionally, 73% of these employers believed students were not fully prepared for the NHS interview process. Therefore, Aston University redeveloped an existing mock interview component to align directly with NHS interview processes. This research aimed to evaluate the effectiveness of the redesigned “Job interview” assessment upon student understanding of their own transferable skills and readiness for future laboratory employment.

**Methods:**

Researchers evaluated the effectiveness of the assessment through a mixed-method approach survey. The survey was launched to students following their completion of the Medical Laboratory Assistant video interview, using the interview software Interview360. The survey sought to identify if after the interview assessment students felt they could demonstrate with examples, using the STAR technique, several key skills sought by employers.

**Results:**

Data was collected from both the 2023-2024 and 2024-2025 final year Biomedical Science cohort. Collected data has been overwhelmingly positive, with 97% of students agreeing that they *“understand the types of questions they would be asked in an NHS interview”* (*p* < 0.0001). In terms of the key skills sought for by employers, 93% of respondents agreed or strongly agreed that they felt they could communicate within a specific situation example their understanding of *“Basic equipment skills”* (*p <* 0.0001) and their *“understanding of laboratory results”* (*p* < 0.0001). Whilst 99% of respondents agreed or strongly agreed that they could demonstrate their “*understanding of laboratory health and safety”* (*p* < 0.0001). Furthermore, respondents reported that the job interview assessment assisted them to demonstrate their transferable skills, including teamwork (81.6%) and organisational skills (71.05%).

**Discussion:**

Student responses identify a positive change to their job interview skills and understanding of the NHS interview process. Here, researchers present the re-modelled graded job interview assessment with the NHS aligned mark scheme, along with four pre-assessment workshops as a process to embed employability into the Biomedical Science curriculum.

## Introduction

Embedding transferable skills into Higher Education undergraduate degrees is known to positively impact graduate outcomes [1, 2]. For Biomedical Science undergraduate courses, Higher Education Institutes (HEIs) use novel pedagogical innovations to embed these skills into their curriculum, which can include case studies [3]; digital tool-kits [4] and final-year research projects [5]. Embedding these skills pave the pathway for a variety of graduate career options, including scientific roles, research and pharmaceutical related roles, teaching, as well as positions in business and non-scientific industries [6]. The importance of these transferable skills is emboldened by graduate employers, who seek skills such as *“teamwork”* and “*communication skills*” [7]. Despite HEIs embedding these skills into the curriculum, a recent survey conducted for the Institute of Biomedical Science (IBMS) identified that 93% of NHS employers do not feel graduates possess the skills required to meet essential criteria for NHS roles [8]. This poses the question, whether students can self-identify these transferable skills being embedded into their curriculum and, more importantly, can communicate examples of these skills identified in their own practice within an interview context.

### Employability Outcomes Addressing the Attainment gap

The ability to communicate transferable skills is of particular importance to students who face attainment gaps, such as students from low socio-economic backgrounds [9] or for students identified as a minority group, such as female students [10] or minority ethnic students [11]. The literature suggests that in addition to academic success, these individuals can be impacted within an interview setting. For example, student success in medical school interviews have been correlated with their familial wealth, with students from lower income families scoring lower than their higher income counterparts [12]. Additionally, cultural differences have been noted across students’ self-presentation within job interviews, with some cultures displaying a difficulty to self-present certain skills, such as identifying a problem to overcome [13]. Therefore, preparing students for graduate interviews at a subject level within the curriculum, could enhance the students’ self-perceptions of their skills which they carry forward with them post-graduation.

The awarding gap between Global Majority students in UK higher education remains a significant challenge. According to national data from the Office for Students (OfS) [14] for the academic year 2021–22, 93% of White students achieved a 2:1 or above, compared to 84% of global majority students. This disparity underscores structural and systemic inequalities that persist across the sector. Within Biomedical Sciences at Aston University, over 90% of current Biomedical Science students are from the most deprived socioeconomic status quartiles one and two [15]. The cohort consists of majority female student (74.88% and 75.88% for the 2023-2024 and 2024-2025 cohort respectively), with a large portion of students identifying as minority ethnic (81.2% and 88.02% for the 2023-2024 and 2024-2025 cohort respectively). This student population comprises a higher-than-average proportion of students from Global Majority backgrounds, but this is reflective of the diversity of the wider Birmingham area and many urban centres in the UK [11]. Yet, despite this diversity and the inclusive ethos that underpins our institution and other institutes with similar cohorts, we continue to observe an awarding gap [16]. This suggests that diversity alone is not enough to close disparities in outcomes; proactive, strategic interventions are essential that must be embedded throughout the curriculum.

### Diversification of Biomedical Science Curriculum

To improve graduate outcomes for BiomedicalScience students, Aston academics have worked closely with the Aston Careers Team and local stakeholders to diversify the curriculum and redesign elements of the employability curriculum. Several opportunities have been developed for students to gain exposure to the graduate job market through the addition of weighted careers focused assessments in each academic year. One example of this, is a poster event where students returning from their placement year share their experiences with first and second-year students. This event showcases a range of different careers, and the disciplines students can complete their registration portfolios in. For final year, alumni have been embedded into the curriculum to showcase a range of careers and individuals, to provide networking opportunities for students looking forward to their post graduate outcomes.

Furthermore, as part of the final year professional development module, Biomedical Science undergraduate students are required to complete a video interview for an NHS Medical Laboratory Assistant (MLA) role within Haematology. Prior to the interview, students are provided with a copy of a real NHS MLA job description and person specification, which they are required to analyse and identify areas of skills and knowledge alignment. For this assessment, students answer and record their responses to four questions using a video interview system [17], mirroring common video interview systems used by graduate employers. Initially, this assessment introduced in 2017 was a pass/fail assessment. However, following work published by Hussain and Hicks (2022) [8], where 95% of surveyed graduate employers in the UK stated that they felt HEIs should do more interview preparation for graduates, this element was revised to become a weighted component in 2023 (worth 20% of the final year Biomedical Science Professional Development module). The shift to the weighted element, reflects our commitment to graduate employability ensuring our graduates can communicate their transferable skills in an interview setting.

Additionally, 73% of pathology employers believed Biomedical Science graduates’ students were not fully prepared for the NHS interview processes [8]. Therefore, Aston Bioscience academics drew on the long-standing relationship with the West Midlands Training Officer (WMTO) group [3] to review the mark scheme for this assessment to reflect real world NHS recruitment processes. Aston academics felt co-ordinating with local graduate recruiters, was of high importance to the redesigned job interview assessment for two reasons. One: working with stakeholders and beneficiaries is at the heart of Aston University’s 2030 strategy [18], and two: due to ∼67% of Aston Biomedical Science students being recruited from the West Midlands. As these students are likely to stay within the area post-graduation they create competition for NHS pathology graduate roles. This competition for NHS pathology jobs is further complicated by there being graduates from other IBMS accredited Biomedical Science degrees in the West Midlands region [19]. Moreover, within the West Midlands region, ∼200 students apply to ∼40 placement positions each year where students would complete the IBMS registration portfolio [3]. As such, the majority of graduates will not have had the opportunity to complete the IBMS registration training portfolio during their degree. Therefore, graduates are unable to work as a Band 5 Biomedical Scientist and will need to secure either a Trainee Biomedical Scientist post or an MLA role where completion of the registration portfolio will be supported [20]. This pushes the competitive nature for a Trainee Biomedical Scientist position within the region higher than that of a Band 5 Biomedical Scientist.

Another strain to the competitive nature of the Trainee Biomedical Scientist position within the West Midlands region is the number of posts that are advertised. Despite the NHS predicted shortfall of 360,000 staff by 2036, which includes laboratory and healthcare staff [21], data powered by LightCast [22], demonstrates that only 321 Trainee Biomedical Scientist posts were advertised throughout the entirety of 2024. Of these 321 Trainee Biomedical Scientist posts, only 31 (∼10%) were located within the West Midlands. By comparison, there were 82 (∼26%) Trainee positions available within the Southwest and 89 (∼28%) in the North West.

With numerous barriers to graduate employment that the current Biomedical Science cohorts face, Aston staff are committed to help students recognise as well as communicate their own transferable skills. Similarly, this is may also help them communicate their subject specific knowledge and laboratory skills. As part of the redesign, students are now provided with four careers workshops prior to the job interview assessment, to assist students in identifying their own skills and how this is paired with the current Biomedical Science labour market within the region of the university. The series of workshops are taught in collaboration between the Aston Careers team and Aston Bioscience academics.

The introduction of these workshops, coupled with the collaborative changes to the job interview assessment mark scheme and grade weighting in 2023, highlights Astons commitment to graduate employability for cohort demographic. The aim of this study was to evaluate the effectiveness of the remodelled job interview assessment by identifying if Biomedical science students could recognise and effectively communicate transferable skills actively sought by employers.

## Materials and Methods

### Preparing for Implementation

**FIGURE 1:**
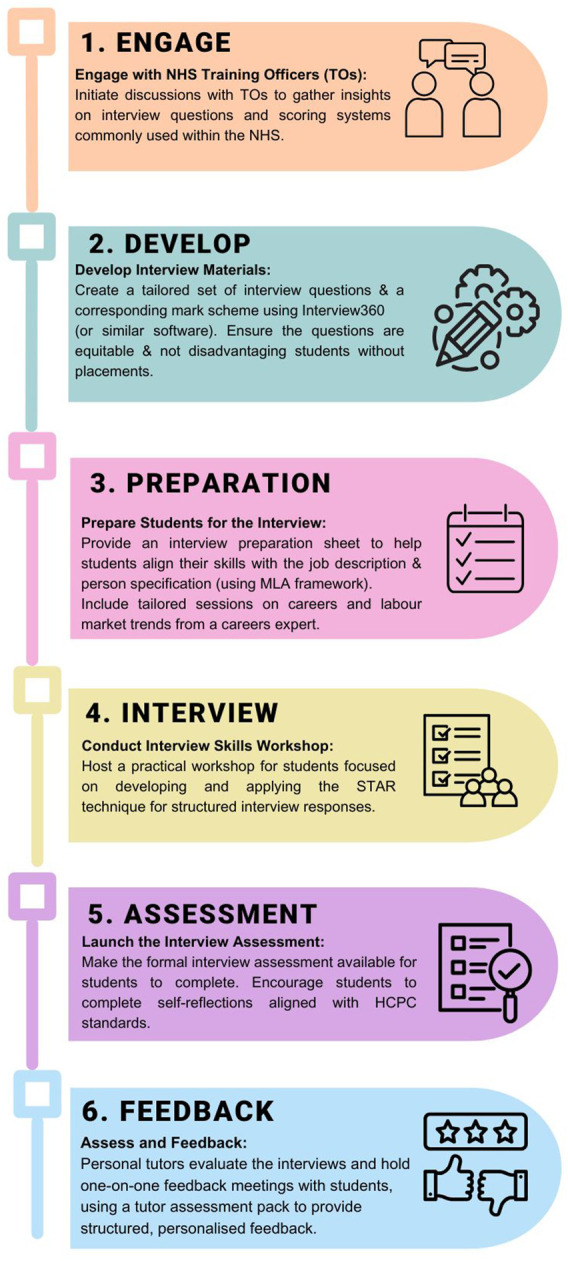
The six-step process involved in creating and delivering a job interview video assessment in the final year undergraduate Biomedical Science curriculum.

Figure 1 details the six-step process involved in creating and delivering the job interview assessment. Each stage of this authentic assessment has been carefully designed to nourish student learning and enable students to be successful at interview [23]. As part of the interview preparation, students receive a series of workshops from the careers consultant which include (i) an introduction to the labour market and career options, (ii) graduate applications, (iii) interview skills (including an introduction to the interview360 software) and (iv) student-led assessment support session. During these workshops, students were encouraged to reflect upon their own experiences and transferable skills which meet the presented Medical Laboratory Assistant (MLA) job description and person specification. In addition, they were also introduced to the STAR (Situation, Task, Action and Result) technique, which is considered best practice when answering interview questions [24].

### Job Interview Assessment Questions

Final year Biomedical Science students were presented with a copy of the Blood Science MLA job description and person specification taken from the NHS jobs website [25]. Following this, students were presented with the job interview assessment worksheet. This worksheet had all four questions they would be asked in the interview and prompted them to reflect on how their own skills meet the criteria requested for MLA post. The four job interview questions presented to final year students were formatted from real NHS interviews, as well as other laboratory-based roles. These were designed by the Aston University Careers team, in collaboration with Aston Bioscience academics and Training Officers within in partnered NHS hospitals. The four questions allowed all final year students to apply their skills to the presented person specification and job description, regardless of if they had completed a placement year or not.

- Question 1: Tell me about yourself–Please provide a brief professional introduction, summarising your motivations for applying for this role and highlighting any key elements of your recent studies, work experiences and extra-curricular activities undertaken.
- Question 2: Please provide an overview of your relevant laboratory and technical experiences.
- Question 3 - Please describe a situation where you have demonstrated NHS values in practice.
- Question 4: Tell us about a time, when you worked well under pressure?

### Student Interview360 Recording and Self-Reflection

For the video interview, students were given 3 weeks to complete the assessment and were assigned a maximum of 3 minutes to record their answer to each of the interview questions using the Interview360 software [17]. Students were able to re-record their answer to each of the questions, allowing them to reflect and improve their answers prior to the final submission. After completion, students were provided with a share link which was placed on the worksheet for the students allocated personal tutor to view their recorded work. Furthermore, following completion of the interview, the worksheet required students to reflect upon their performance with the simple reflection technique of *“what went well?”* and *“even better if…”* prompts [26], before submitting the entire worksheet to their tutor.

### Collecting Student Feedback and Analysis of Results

Student experiences of the job interview assessment were collected post submission, through completion of an online questionnaire [27]. Students were invited to participate in the study by the link to the online questionnaire being made available at the start of the next teaching session and via the announcement function on the virtual learning environment. A mixed method approach was used in the questionnaire, consisting of five-point Likert-scale responses (*4 = strongly agree, 3 = agree, 2 = disagree, 1 = strongly disagree, and 0 = neither agree or disagree*) and a series of open-ended questions. Free-text questions allowed students to list the transferable skills sought for by employers, which the job interview assessment has highlighted to them that they possess. The results were analysed both quantitatively and qualitatively.

### Statistical Analysis

Quantitative data gathered from the survey is reported as the percentage of respondents who agreed or strongly agreed with each statement. IBM-SPSS Statistics version 26 [28] was used to perform a chi-squared analysis to test whether there was a difference in within both cohorts’ collective responses to each of the questions. For 3 degrees of freedom, a chi-square value of 7.815 with statistical significance set at *p* ≤ 0.05. Free-text responses were analysed using thematic analysis [29].

## Results

Following the job interview assessment, students were asked to reflect on their interview skills and identified transferable skills post-assessment completion. The survey was launched to both the 2023-2024 and 2024-2025 student cohorts. Response rates varied between the two cohorts, with a total of 25/98 responses in the 2023-2024 cohort (25% response rate) and 51/109 responses in the 2024-2025 cohort (47% response rate). Therefore, over two academic years, a total of 76 students (37%) responded to the online survey.

### Overall Interview Skills

**FIGURE 2:**
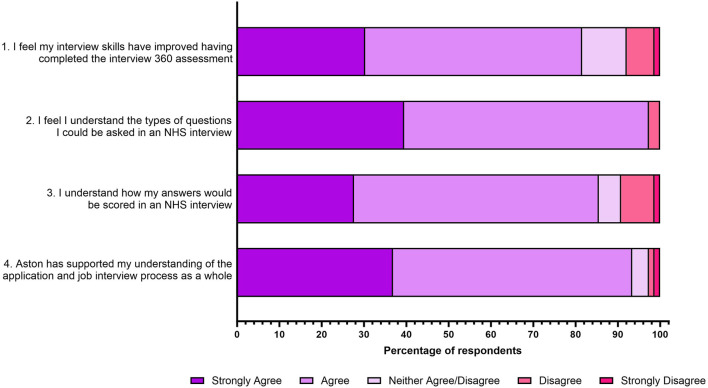
Student responses to four statements relating to increased overall interview skills post interview assessment. The responses to the five-point Likert scale for each statement is shown as a percentage.

Responses to four questions regarding interview skills are shown in Figure 2 below. A total of 82% of respondents “*strongly agreed or agreed*” that their interview skills had improved after completing the interview 360 assessment (statement 1), with 97% of respondents reporting that they understood the types of questions that could be asked in an NHS interview (statement 2). Additionally, 86% of respondents “*strongly agreed or agreed*” that they understood how their answers would be scored in an NHS interview (statement 3), with 93% reporting that they felt supported by the university in understanding the application and interview process (statement 4).

### Employer Specific Skills

**FIGURE 3:**
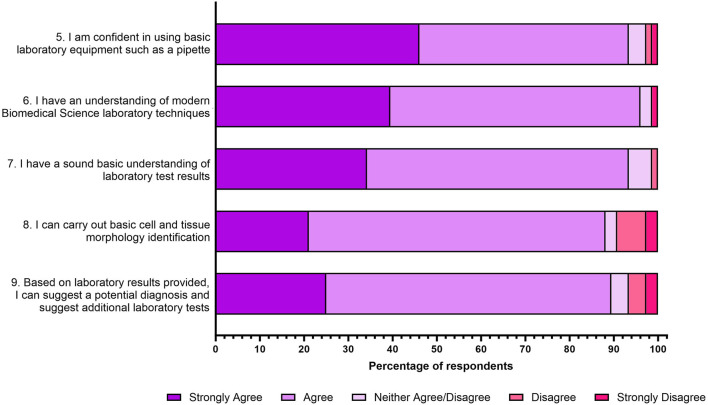
Student responses to five statements relating to self-identification of NHS graduate employer sought for skills post interview assessment. The responses to the five-point Likert scale for each statement is shown as a percentage.

Next, participants were asked whether after completing the job interview360 assessment, they felt confident in their skills actively sought by NHS employers. As shown in Figure 3, 93% of respondents reported feeling that they were confident in using basic laboratory equipment such as a pipette (statement 5), with 96% of respondents selecting “*strongly agree or agree”* that they had an understanding of modern Biomedical Science laboratory techniques (statement 6). A total of 93% of respondents reported having a sound understanding of laboratory test results (statement 7), with 88% of respondents either “*strongly agreeing or agreeing*” that they can carry out basic cell and tissue morphology identification (statement 8). When asked whether based on test results they could suggest a potential diagnosis and recommend additional laboratory tests, 90% of respondents *“strongly agreed or agreed”* with this statement (statement 9).

### Overall Laboratory Readiness and NHS Values

**FIGURE 4:**
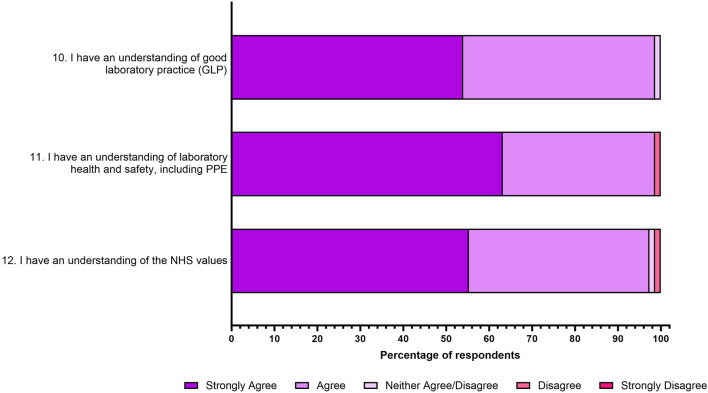
Student responses to three statements relating to NHS laboratory readiness post interview assessment. The responses to the five-point Likert scale for each statement is shown as a percentage.

Students were then presented with three statements relating to laboratory readiness and NHS values. As shown in Figure 4, 99% of respondents *“strongly agreed or agreed”* that they have an understanding of good laboratory practice (statement 10), as well as laboratory health and safety (statement 11). Finally, 97% of respondents *“strongly agreed or agreed”* that they have an understanding of the NHS values (statement 12).

### Job Interview Significantly Enhanced Student Understanding of the NHS Interview Process

In general, students overwhelmingly agreed that they benefited from engaging with the job interview assessment, specifically helping them to improve their interview skills, their understanding of vocational-technical skills and their understanding of NHS values (Figures 2–4). A chi-squared test was conducted to investigate how much the observed data deviates from what would be expected under the assumption that the variables have no relationship between them (Table 1). For 3 degrees of freedom, a chi-square value of 7.815 is the cut-off for statistical significance (*p* < 0.05). Table 1 highlights that almost all chi-square values are above the threshold, and it can therefore be determined that all associations exhibit strong evidence against independence. However, students were asked if they felt if they had a good understanding of laboratory practice following the job interview assessment and this variable exhibited a non-significance (chi-square value 0.842 and *p* value = 0.359).

**TABLE 1:** Descriptive statistical analysis of survey questions presented to two cohorts of final year Biomedical Science undergraduate, using a chi-squared test to investigate the deviation of observed data from what would be expected under the assumption that the variables have no relationship between them.

Overall Interview Skills	N	Mean	Std. Deviation	Minimum	Maximum	Chi-Square	df	Significance
I feel my interview skills have improved having completed the interview 360 assessment	76	3.3158	0.65748	1	4	56.000a	3	**0.0001**
I feel I understand the types of questions I could be asked in an NHS interview	76	3.3684	0.5377	2	4	36.105b	2	**0.0001**
I understand how my answers would be scored in an NHS interview	76	3.2237	0.64495	1	4	60.737a	3	**0.0001**
Aston has supported my understanding of the application and job interview process as a whole	76	3.3684	0.5852	1	4	72.000a	3	**0.0001**

Employer Specific Skills	N	Mean	Std. Deviation	Minimum	Maximum	Chi-Square	df	Significance
I am confident in using basic laboratory equipment such as a pipette	76	3.4605	0.59868	1	4	68.316a	3	**0.0001**
I have an understanding of modern Biomedical Science laboratory techniques	76	3.3947	0.56754	1	4	37.447b	2	**0.0001**
I have a sound basic understanding of laboratory test results	76	3.3816	0.51555	2	4	39.500b	2	**0.0001**
I can carry out basic cell and tissue morphology identification	76	3.1184	0.63176	1	4	79.474a	3	**0.0001**
Based on laboratory results I can suggest a potential diagnosis and suggest additional laboratory tests	76	3.1974	0.63287	1	4	76.526a	3	**0.0001**

Overall Laboratory Skills	N	Mean	Std. Deviation	Minimum	Maximum	Chi-Square	df	Significance
I have an understanding of good laboratory practice	76	3.5526	0.50053	3	4	.842c	1	0.359
I have an understanding of laboratory health and safety, including PPE	76	3.6184	0.51555	2	4	43.763b	2	**0.0001**
I have an understanding of the NHS values	76	3.5526	0.52649	2	4	37.447b	2	**0.0001**

The bold values are statistically significant.

### Thematic Analysis of Self-Identified Transferable Skills

**FIGURE 5:**
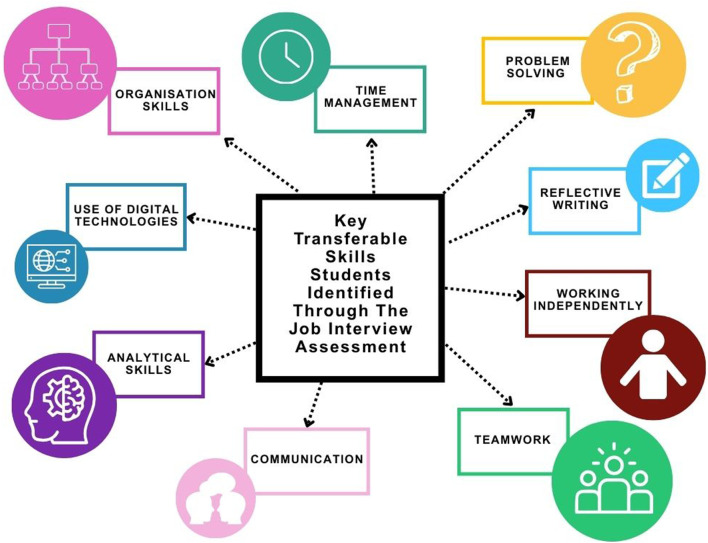
A thematic analysis displaying the key transferable skills students identified through completion of the job interview assessment. Nine major themes were identified, and the occurrence of each theme is represented by the size of each circle in the schematic. Several students responses contained more than one theme.

Furthermore, in the post-interview survey, students were asked to self-identify the key transferable skills they believed the job interview assessment helped them communicate (Figure 5). A total of 100% of respondents (*n* = 76) answered this question. The most frequently reported skills included teamwork (82%), organisation skills (71%), working independently (67%), problem solving (68%), time management (68%), analytical skills (58%), communication (47%), reflective writing (47%) and use of digital technologies (41%). Students were encouraged to select multiple transferable skills, they developed and were able to demonstrate in an interview setting.

### Thematic Analysis of Free-Text Options

Students were given the opportunity to provide free-text responses regarding the job interview assessment. Twenty students (26% of respondents) provided additional comments. Nine of these twenty students included comments relating to the effectiveness of the interview process such as:

“A couple days after completing this task I had a job interview within the NHS and this assignment REALLY helped me. I was able to use the STAR technique and reflect on my responses. I also had a great understanding of NHS values and the interviewers were extremely impressed with my knowledge and answers. They also liked that I reflected on each of my answers rather than just giving generic responses.”

“It was amazing, and it did boost my confidence for my upcoming job interviews”

“It helped highlight the areas of an interview where I would have struggled had I not prepared. I found this particularly useful because now I can keep that in mind when I do get an interview.”

Furthermore, the free-text responses of eleven of these twenty students suggested future improvements to the job interview assessment. Students felt these changes would benefit them even further than the job interview currently does, with responses including:

“I feel like the questions shouldn't be made available to us in advance as the questions would be unknown on the real day.”

“During the pre-assessment workshops, could we have an example of a placement student and an example of a non-placement student. To provide more support and how we can answer the questions with transferable skills.”

“The interviews contain more laboratory technical questions, such as 'What happens when blood samples are left out of the fridge?' or 'What are the risks of handling a biological hazard and how to reduce those risks?'.”

## Discussion

In the evolving landscape of healthcare and diagnostics, the role of the Biomedical Scientist is becoming increasingly complex and central to patient care. Hussain and Hicks, 2022 [8] demonstrated that 93% of NHS graduate employers felt students did not possess the skills for graduate NHS roles. Alongside technical laboratory skills, employers, especially within the NHS and associated healthcare sectors are seeking graduates who possess a broader range of professional skills, including communication, teamwork, critical thinking, and interview readiness [30]. As such, embedding a structured employability curriculum within Biomedical Science programmes is no longer optional; it is essential. Conversely, research from HEIs demonstrates these transferable skills are embedded within the Biomedical Science curriculum [4, 29, 31, 32]. This suggests that students struggle to self-identify these transferable skills in an interview setting, despite having the opportunity to develop these skills in the curriculum. Furthermore, Hussain and Hicks, 2022 [8], identified 95% of employers believed HEIs needed to do more to prepare students for interviews. Aston University have delivered employability workshops and assessments within the curriculum since 2017. To further emphasise the importance of these skills, the final year interview assessment was remodelled in collaboration with regional NHS laboratory Training Officers and the Aston Careers team to mirror the NHS interview process. This study aimed to identify if these assessment changes improved students’ self-recognition of their skills and empowered them with the ability to communicate these effectively.

### Development of Interview Skills

The importance of fostering interview skills within undergraduate curriculum has been reported for different courses [33, 34]. Particularly, for Biomedical Science undergraduates as the graduate market is ever-changing [35]. Furthermore, 73% of surveyed NHS graduate employers reported that graduates were not prepared for NHS interviews [8]. Suggested best practice for the development of interview skills is through mock interviews with the use of pre-interview class discussions [36, 37]. This study demonstrates with the addition of four pre-assessment workshops prior to a mock video interview students perceived interview skills improved (82% *strongly agreed or agreed*; *p <* 0.0001). The interview questions and associated mark scheme were created in collaboration with local NHS graduate employers. Students reported feeling confident in understanding the types of questions featured in NHS interviews (97% *strongly agreed or agreed*; *p* < 0.0001) and students understood how these questions would be scored (86% *strongly agreed or agreed*; *p* < 0.0001).

Whilst the importance of university careers teams has been reported across the UK [34], it has been shown that students underutilise this precious resource [38, 39]. Following the addition of the pre-assessment workshops, students are presented with a direct link to the Aston University Careers team within their final year of study. Respondents within this study reported that this link during the interview assessment made them feel supported by the university in understanding the application and interview process (93% *strongly agreed or agreed*; *p* < 0.0001). In addition, the Aston Careers team have noted that the final year Biomedical Science cohort are the most engaged course from the College of Health and Life Sciences. Whilst increased engagement cannot be directly attributed to this specific assessment, in 2023/2024, 65% of the final year Biomedical Science cohort booked one or more individual meetings with the Aston Careers team. A similar trend was seen for the final year 2024/2025 cohort, where 64% of the cohort booked one or more meetings. This suggest that having the Careers team led sessions woven into the core curriculum encourages students’ engagement with this valuable resource.

### Employer Sought for Skills

Many HEIs often place emphasis on grades, whereas employers place emphasis on competencies and transferable skills [40]. Therefore, it is important for HEIs to understand the skills that graduate employers seek to prepare their graduates for the working world [41]. Hussain and Hicks, 2022 [8] highlighted six top skills that NHS graduate employers were seeking, which Aston staff feel are already available through various learning opportunities within the Aston Bioscience’s curriculum. This research suggests that students cannot articulate these skills in interviews therefore, students were asked whether they could communicate how they demonstrate these skills using the STAR interview technique following the assessment [25] (Figure 3).

Following the tailored interview assessment, students reported increased confidence in communicating an example which demonstrates their use of basic laboratory equipment (96% *strongly agreed or agreed*; *p* < 0.0001); understanding of laboratory test results (93% *strongly agreed or agreed; p* < 0.0001); carrying out basic cell and tissue morphology identification (88% *strongly agreed or agreed; p* < 0.0001) and their ability to suggest a potential diagnosis and recommend additional laboratory tests (90% *strongly agreed or agreed*; *p* < 0.0001). This mirrored four of the skills sought for by NHS graduate employers [8].

Not all final year Biomedical Science students, have undertaken an NHS placement year or work part-time within an NHS laboratory. Therefore, to make the assessment accessible to all students the *“Experience of an NHS laboratory”* was changed within the interview to highlight student *“understanding of modern Biomedical Science laboratory techniques”* of which 96% *strongly agreed or agreed* (*p <* 0.0001). The final skill identified by Hussain and Hicks, 2022 [8] “*Interpretation of internal quality control (IQCs)*” was not included in this assessment, as IQCs, such as Levy-Jennings plots [42], are assessed through the Professional Skills exam later in the academic year.

Hussain and Hicks, 2022 [8] also highlighted that 64% of NHS graduate employers sought for a range of personal skills. It is important HEIs are aware of the transferable skills employers seek and embed opportunities to develop these within the curriculum [43]. Through the free-text options, students were able to report any transferable skills that they felt they could communicate at an interview following the assessment. All respondents that completed this question highlighted nine transferable skills (Figure 5). Notably, time management was identified by 68% which was also highlighted by Hussain and Hicks, 2022 [8] as being important to NHS graduate employers. Furthermore, the two most prominent transferable skills identified within this study were teamwork (82%) and organisation skills (71%). Both of these skills appear within the job person specification for “Trainee Biomedical Scientist” and “Medical Laboratory Assistant” roles advertised in 2024 within the UK, using data available from Lightcast [22]. Within this study, 47% of students identified communication as a key transferable skill that they had developed. From Lightcast [22] data, analysing the forementioned job role advertisements in 2024, one of the most sought-after skills for both roles, was communication, further highlighting the alignment with regional and national demands.

### Overall Laboratory and HCPC Readiness

Approximately 88 universities offer undergraduate (BSc) Biomedical Science courses [44] and currently 64 of these are accredited by the IBMS [19]. Like other IBMS accredited courses, Aston University aims to prepare the graduates for registration with the Health and Care Professions Council (HCPC) [45]. This requires graduates to meet the Biomedical Scientist Standards of Proficiency [45]. Two of these proficiencies are “*14.6 understand the application of principles of good laboratory practice”* and “*14.3 work safely, including being able to select appropriate hazard control and risk management”.* These skills are also required by educational institutes in the Standards of Education and Training [46]. As such, this research sought to identify if students felt that they could identify these skills within themselves following the job interview assessment (Figure 4).

Overwhelmingly, students *‘strongly agreed or agreed*’ that they could identify their own understanding of good laboratory practice (99%; *p* = 0.359) and their understanding of laboratory health and safety, including PPE (99%; *p* < 0.0001). Interestingly, of all the survey questions, the students understanding of good laboratory practice is the only one that does not meet statistical significance, which could be in part that no students *‘disagreed or strongly disagreed*’ with the statement. This may have occurred as 2 weeks prior to the interview assessment being launched, students participated in a laboratory practical competency assessment and therefore felt they already knew how to demonstrate this skill. Indeed, a large portion of students used the laboratory competency assessment as part of their STAR answer for interview question 2 “*Please provide an overview of your relevant laboratory and technical experiences”.*

Finally, students were asked if they felt following the interview assessment, they understand how to communicate the NHS values with 97% *‘strongly agreeing or agreeing*’ (*p* < 0.0001) with this statement. Researchers chose to include this question as the NHS is the largest employer of IBMS accredited Biomedical Scientists [47], and the six core values are at the heart of the NHS constitution [48]. Therefore, this seemed an important point for students to be able to communicate effectively during interviews, which is why the Aston Bioscience team embedded these in during question 3 *“Please describe a situation where you have demonstrated NHS values in practice*”. As respondents have strongly identified this skill after the job interview assessment, this should help these graduates in future NHS employment. Respondents who have since undertaken NHS interviews following graduation have provided feedback to the Aston Careers team that the job interview assessment questions mirrored those asked in NHS interviews. One such comment stated, “*After completing the job interview assessment yesterday, today I had a real NHS interview, and the questions were EXACTLY the same*”.

### Future Work and Study Limitations

During this study, students used the free-text options to suggest future improvements to the job interview assessment. For assessments to fit student’s needs, it is important for HEIs to take on this feedback and make the changes where possible [49]. Several requested improvements are currently within the control of Aston Biosciences to include in the launch next academic year (2025-2026), including the addition of technical questions such as *‘how to label samples correctly’* and providing student exemplars with different levels of work experience (placement vs. non-placement). However, some of these improvements can be difficult to implicate due to student cohort size, such as allowing students to select a “job” that they mock interview for. This could particularly prove challenging due to the wide range of careers a graduate of Biomedical Science could enter into [6].

In terms of study limitations, the overall response rate for both cohorts were 25% (2023-2024) and 47% (2024-2025). This does not deviate greatly from the average response rate for similar surveys that usually generate a 30%–40% uptake [50]. However, researchers acknowledge that this could be improved in future work. Literature suggests that a monetary incentive can improve the response rate [51]. Although a student from each cohort was allocated a £50 voucher at random, perhaps a smaller monetary incentive could be considered for every student who replies. Another way to potentially improve response rates in future work is the concept of a postpaid incentive which was only released after a certain number of responses are met [52].

### Application of Novel Assessment to Other Institutions

At Aston, the addition of the mock interview has significantly improved students’ ability to communicate key skills sought for by employers as demonstrated by this study. Furthermore, since the changes made to the grade weighting for the mock interview and the inclusion of four pre-assessment workshops, the graduate outcomes of Biomedical Science students have been increased by ∼8% on The Times National League table [53]. While researchers cannot imply causation, the Times 2025 graduate prospects score of 87% is currently the highest the Biomedical Science course at Aston has seen in the last 5 years. This places Aston Biosciences at 24th in the UK out of 95 institutions but 1^st^ within the West Midlands Region for Biomedical Science [44].

**FIGURE 6:**
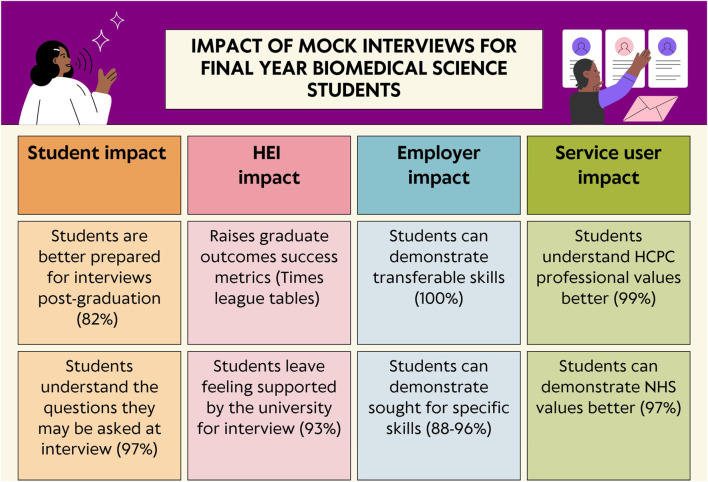
An infographic demonstrating the impact of the mock interview assessment on the students, the HEI, the employer and ultimately, the pathology service user.

The results presented, demonstrate a clear benefit of embedding mock interviews within the Biomedical Science curriculum to widen post-graduation participation. Researchers have already presented the six-step process involved within the assessment set up within Figure 1. Given the challenges within higher education that often limit students’ access to placements and experiences beyond the core Biomedical Science curriculum, the Aston team strongly advocates for the integration of this assessment model across other institutions to enhance student development and employability. Doing so not only enhances student development and career readiness, but also addresses critical workforce gaps in the NHS. By better preparing graduates for clinical and diagnostic roles, universities can contribute to a more resilient healthcare system, ultimately improving outcomes for patients who depend on timely, accurate biomedical support. As suggested by Hussain and Hicks [8] and the Advanced HE [54] The impact of the mock-interview can be of benefit to the student, HEIs, employers and ultimately the service users (Figure 6).

While this research demonstrates that the framework of four pre-assessment workshops and the accompanying graded mock interview has been successful, the researchers acknowledge potential limitations for other higher education institutions seeking to implement a similar mock interview component within their programmes. It is not possible for students to self-recall skills if they have not had the opportunity to gain the transferable skills to begin with [3, 4, 29, 32]. Furthermore, within the UK it is noted that 45% of HEIs do not have any pre-employment with their modules [8]. These HEIs may encounter more limitations if they decide to implement the job interview framework into their own programmes, as there is not a logical flow throughout all 3 years. In addition, there may also be cost restraints if the HEI does not already have access to the Interview360 software [17], but this could be overcome with the use of video software accessible to students, such as recordings on mobile phones.

However, these limitations can be overcome with the shift to employability-focused education, which not only enhances students’ readiness for the workplace but also directly contributes to workforce sustainability within the NHS. A well-designed employability curriculum can improve student confidence, support career planning, and increase access to placement opportunities, all of which are linked to higher graduate outcomes and reduced awarding gaps across diverse student cohorts.

To better prepare graduate and improve social mobility, Biomedical Science courses, should consider the following strategies:

1. Integrate employability across the curriculum: Development of credit-bearing modules/elements that are focused on career development, professional identity, and NHS recruitment processes. Assessments such CV writing, reflective practice, and interview skills can enhance students’ ability to professionally communicate their transferable skills. A scaffolded approach reinforces continuous professional development.
2. Engage career teams in curriculum design and delivery: The successful integration of employability content into academic programmes requires a collaborative, team-based approach. Careers professionals bring valuable expertise in labour market trends, employer expectations, and recruitment practices, and their early involvement in curriculum design can ensure that employability elements are both relevant and impactful. By working in partnership with academic staff, careers consultants can provide tailored insights and practical strategies that align course content with the evolving demands of the biomedical science sector.
3. Industry and NHS engagement: Collaborate with NHS laboratories, IBMS representatives, and placement providers to co-design assessments ensuring students understand current workforce expectations.
4. Placement preparation pathways: By engaging with stakeholders and partaking in structured placement processes like the West Midlands Applied BMS placement process, universities can create a pathway to enable students to gain valuable experience [3]. To prepare students for these roles, courses need to offer structured pre-placement support, including application guidance, interview practice, and professional behavior workshops. This is especially valuable in addressing barriers faced by underrepresented student groups.
5. Monitoring and evaluation: The graduate workforce is dynamic and always evolving. Therefore, employability measures and interventions require regular reviews using student feedback, progression data, and post-graduation employment metrics to ensure relevance and impact. An example of this is changing academic practice considering increased use of Artificial Intelligence (AI) by job applicants to create CVs and employers to screen candidates [55].

By embedding a well-structured employability curriculum, Biomedical Science programmes can better prepare students for the challenges of the modern healthcare environment, strengthen links with employers, and contribute to more equitable and sustainable graduate outcomes across the sector. At present, accredited Biomedical Science courses are offered by 64 universities across the UK [19], but 45% do not have any employability within their curriculum [8]. Courses that are accredited by the IBMS are required to demonstrate evidence that their students meet core competencies upon graduation, currently there are no benchmarks statement specifically related to employability. We believe the addition of employability related criteria to these IBMS competencies, would widen participation post-graduation and increase social mobility.

## Conclusion

This study aimed to evaluate the effectiveness and relevance of the redesigned interview assessment, and the findings indicate that it has provided a meaningful and beneficial learning experience for students, supporting its suitability for the intended purpose. The research presented addresses the clear need for HEIs to better prepare students for NHS interviews and achieve graduate outcomes. The work presented was conducted in direct response to Hussain and Hicks, 2022 [8], suggesting ways to address the solutions within their research, one example being mock interviews. Whilst Aston already had a mock interview assessment, the pass/fail weighting did not stress the importance of the assessment. Therefore, a collaboration between Aston Bioscience academics, the Aston Careers team and local NHS graduate employers created a grade mock interview assessment to be part of the final year Professional Development for Biomedical Scientists. This directly supports other research, which suggests employability should be embedded into the Biomedical Science curriculum rather than being an additional non-assessed element [2].

## Summary Table

### What Is Known About This Subject

- NHS graduate employers feel that students are not prepared for NHS interviews post-graduation.
- Transferable skills are increasingly sought for by employers over grade classifications.
- Students cannot always self-identify their own transferable skills in interview settings with no prior training.

### What This Paper Adds

- Provides a pedagogical framework to embed mock interviews into the Biomedical Science curriculum.
- The interview assessment encourages students to self-identify their transferable skills and communicate these effectively.
- The interview assessment enhances students’ graduate outcomes and encourages engagement with the careers team.

## Concluding Statement

This work represents an advance in biomedical science because the mock interview assessment assists student’s ability to communicate skills effectively to employers.

## Data Availability

The raw data supporting the conclusions of this article will be made available by the authors, without undue reservation.
